# Provider Adoption of mHealth in Rural Patient Care: Web-Based Survey Study

**DOI:** 10.2196/55443

**Published:** 2024-06-24

**Authors:** Bryan P Weichelt, Rick Burke, Burney Kieke, Matt Pilz, Neel Shimpi

**Affiliations:** 1 National Farm Medicine Center Marshfield Clinic Research Institute Marshfield, WI United States; 2 Center for Clinical Epidemiology & Population Health Marshfield Clinic Research Institute Marshfield, WI United States; 3 American Dental Association Chicago, IL United States

**Keywords:** mHealth, clinician, physician, rural, patient, mobile, health care, adoption, attitude, attitudes, opinion, perception, perceptions, perspective, perspectives, acceptance, mobile health, app, apps, provider, providers, physicians, survey, surveys, barrier, barriers, digital health

## Abstract

**Background:**

Physicians and patient-facing caregivers have increasingly used mobile health (mHealth) technologies in the past several years, accelerating during the COVID-19 pandemic. However, barriers and feedback surrounding adoption remain relatively understudied and varied across health systems, particularly in rural areas.

**Objective:**

This study aims to identify provider adoption, attitudes, and barriers toward mHealth in a large, multisite, rural US health care system. We investigated (1) mHealth apps that providers use for their own benefit and (2) mHealth apps that a provider uses in conjunction with a patient.

**Methods:**

We surveyed all patient-seeing providers within the Marshfield Clinic Health System with a brief, 16-item, web-based survey assessing attitudes toward mHealth, adoption of these technologies, and perceived barriers faced by providers, their peers, and the institution. Survey results were summarized via descriptive statistics, with log-binomial regression and accompanying pairwise analyses, using Kruskal-Wallis and Jonckheere-Terpstra tests for significance, respectively. Respondents were grouped by reported clinical role and specialty.

**Results:**

We received a 38% (n/N=916/2410) response rate, with 60.7% (n=556) of those sufficiently complete for analyses. Roughly 54.1% (n=301) of respondents reported mHealth use, primarily around decision-making and supplemental information, with use differing based on provider role and years of experience. Self-reported barriers to using mHealth included a lack of knowledge and time to study mHealth technologies. Providers also reported concerns about patients’ internet access and the complexity of mHealth apps to adequately use mHealth technologies. Providers believed the health system’s barriers were largely privacy, confidentiality, and legal review concerns.

**Conclusions:**

These findings echo similar studies in other health systems, surrounding providers’ lack of time and concerns over privacy and confidentiality of patient data. Providers emphasized concerns over the complexity of these technologies for their patients and concerns over patients’ internet access to fully use mHealth in their delivery of care.

## Introduction

Increased technological and medical advancements have naturally led to the intersection of these 2 fields of study, commonly known as mobile health (mHealth) [1]. mHealth has been defined as “mobile computing, medical sensor, and communication technologies for healthcare” and has seen increasing adoption in recent years, particularly following the shift to virtual health delivery during the COVID-19 pandemic [1-3]. However, given privacy concerns, institutional hesitancy, and the wide array of programs and devices available, adoption and use of mHealth have been mixed [4].

The 2 prevailing mobile platforms include Android and iOS, which collectively comprise more than 99% of mobile use today on phones, tablets, and wearable devices [5]. Traditionally, the development of mobile apps required maintaining separate codebases and expertise per platform. Advances in web technologies, coupled with the establishment of these 2 universal operating systems, have ushered in new and dynamically evolving cross-platform solutions. Vastly expanded broadband cellular networks have simultaneously led to a surge in mobile accessibility, with 95% of the globe reaching 3G coverage as of 2022 and 80% obtaining 4G or faster speeds, including throughout rural regions [6].

To streamline development resources while maximizing user reach, cross-platform frameworks have become dominant across all sectors of mobile apps, including mHealth [7]. These libraries leverage existing technologies, often derivatives of web languages such as HTML, cascading style sheets, and JavaScript, while seamlessly interfacing with the native capabilities of each platform. Two of the most popular architectures for modern cross-platform apps include React Native (Meta Platforms) and Flutter (Google) [8]. Similarly, 2 popular hybrid solutions include Cordova and Capacitor, which can efficiently embed existing websites into native web views to achieve familiar native app behavior.

Emerging app-connected wearables—smartwatches, eyewear, earwear, and clothing—synergize with cross-platform apps to offer new ways of interacting with consumers and patients. Adaptation of wearable technologies has boomed in recent years, with a projected 1 billion circulated wearables in 2022 compared with 325 million in 2016 [9]. Android and iOS smartwatches offer core health initiatives, with sensors and apps that can automatically analyze heart health, blood oxygen, sleep cycles, and fitness. This innovation is rapidly superseding traditional life alert functionality with recent developments including fall detection, automatic location-aware emergency dialing, predictive warnings of heart arrhythmia, and reported seamless syncing of medical records [10].

Many promising new use cases of wearable tech in the mHealth industry are emerging after years of research and pilot studies. For example, after 12 years of testing, Apple has proofed a noninvasive continuous blood glucose monitor system that uses silicon photonics and optical spectroscopy, which is expected to be miniaturized into a common watch-sized wearable within 3 years for consumer use [11]. Manufacturers have also continued exploring augmented reality medical applications for eyewear and are working on adaptive lens adjustment technologies, which would dynamically adjust to one’s eyesight with no prescription lens required. Other types of wearable devices are continuously being tested, including ones that can monitor saliva or tear gland fluids to detect eye or oral diseases, among other medical conditions [12].

These advances in technologies and applications have moved at incredible speeds, most often ahead of health systems’ and providers’ organizational abilities or individual preparedness to adopt, test, and implement for their own use or use with patients. Nonetheless, health care providers can and do leverage available advances in medical technologies for the benefit of the patient, and we would fully expect that mHealth apps and wearable technologies are no exception.

To better understand the current environment of mHealth adoption and barriers among rural providers and patients, we sought to further explore two key topics in this study: (1) apps that providers use for their own benefit and (2) apps that a provider uses in conjunction with a patient.

Khatun et al originally described a conceptual model for mhealth readiness through the lens of a health workforce in rural Bangladesh [13]. The model was later advanced and refined by Weichelt et al in 2019, furthering discussions of the interplay of rural patients, clinicians, and their organizations in mHealth adoption [14,15]. This prior research found that the organization plays a role in impacting providers’ and patients’ adoption of mHealth; however, we need to first gain a deeper understanding of providers’ current levels of adoption and familiarity and awareness with these new technologies.

This line of research, beginning with an assessment and inventory of mHealth adoption, is essential for the future of health care delivery. Marshfield Clinic Health System (MCHS) is a predominantly rural health system with patients scattered across northern Wisconsin, the Upper Peninsula of Michigan, and beyond [16]. While well positioned to test and deploy new and innovative technologies in the broad field of mHealth, leadership first needs to gain a deeper understanding of the system’s provider and patient needs, desires, and current use. Therefore, we conducted a survey of all patient-seeing providers within MCHS to identify mHealth adoption, attitudes, and perceived barriers to use.

## Methods

### Data Collection

In July of 2020, we emailed a survey to 2410 MCHS providers via an information systems–supplied “MCHS Providers” email list. The survey was designed to assess providers’ motivators and barriers to the adoption of mHealth technologies in patient care. The survey was open and available from July 21 to August 31 (6 weeks), with 2 reminders sent every 2 weeks.

Instrument design and line of inquiry leveraged previous work by this research team, including the previously published conceptual model for assessing necessary conditions for rural health care’s mHealth readiness, with an emphasis on clinician-perceived barriers. Providers were asked about mHealth use, both personal and with patients, as well as personal, perceived colleague, patient, and institutional barriers to mHealth adoption. Providers were also asked about the perceived COVID-19 impact on mHealth use and anticipated future mHealth use after the pandemic subsided.

### Incentives

Participants were presented with the option of selecting one of five local nonprofits to receive a US $10 donation for their voluntary participation in the survey. We distributed our full budgeted allotment of US $2800 as chosen by the research participants. No other incentives were offered during the study.

### Ethical Considerations

The project submission was evaluated by the Marshfield Clinic institutional review board. It was determined that the activity as described does not meet the definition of human participant research, and no further institutional review board action was needed.

### Analyses

**Figure 1:**
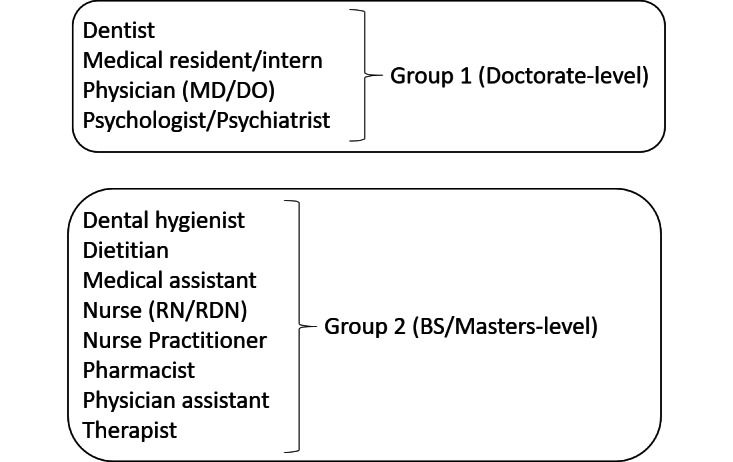
Grouping of provider roles. RDN: Registered Dietitian Nutritionist; RN: registered nurse.

Due to the small number of responses in some niche roles and specialties, participants’ roles were grouped into 2 categories based on education and degree level (Figure 1). Provider specialty was also compartmentalized into 9 categories, mirroring the distribution of specialties across MCHS.

We used log-binomial regression to analyze survey questions with dichotomous (yes or no) responses [17]. Specifically, we fit univariable models where the dependent variable was the dichotomous response and the independent variable was either provider role, provider specialty, or number of years in practice (7 categories: 0-5, 6-10, 11-15, 16-20, 21-25, 26-30, and >30 years). We assessed the overall statistical significance of the independent variable and proceeded with pairwise comparisons versus a referent category when warranted (ie, when the *P* value for the overall effect was ≤.05). BS and MS-level providers, family medicine, and 16-20 years in practice were the referent categories in the calculations. Since provider roles had 2 categories, corresponding pairwise comparisons were unnecessary (redundant).

A similar general strategy was used for Likert-scaled survey questions (ie, assessment of the overall statistical significance of the independent variable followed by pairwise comparisons vs a referent category when warranted). The Kruskal-Wallis test was used to evaluate overall significance and the Jonckheere-Terpstra test for pairwise comparisons [18,19]. The same independent variables were examined.

## Results

### Overview

We received a total of 916 responses (38% response rate), of which 556 (60.7%) responses were sufficiently complete to be included in the statistical analyses. Of these responses, 301 (54.1%) participants reported using health-related apps on their phone or tablet. The most common purposes for these apps were use as informational resources (234/301, 77.7%) and for decision-making (180/301, 59.8%). Providers who used mHealth with patients (202/556, 36.3%) reported doing so primarily for exercise and activity monitoring (105/202, 52%), and enhancing patients’ experiences via the My Marshfield Clinic app (105/202, 52%), an in-house app that allows patients to schedule appointments, view lab results, message providers, etc. Those who do not use health-related apps (255/556, 45.9%) stated their primary reasons as inadequate information available on the use of such apps (59/255, 23.1%), not having enough time to use the apps (55/255, 21.6%), or being unsure of the organization’s attitudes toward mHealth (59/255, 23.1%). The most common barriers to mHealth adoption cited by providers were a lack of both knowledge about mHealth technologies (293/556, 52.7%) and time (201/556, 36.2%), as well as being unsure of their patients’ access to reliable internet services (171/556, 30.8%). These same concerns arose when we asked respondents which barriers they thought other providers had surrounding mHealth (319/556, 57.4%; 283/556, 50.9%; and 163/556, 29.3%). Perceived organizational barriers to clinicians using mHealth in their practices were primarily concerns related to confidentiality (313/556, 56.3%) and mHealth technologies being too complicated for patients (288/556, 51.8%). Overall, however, providers had a favorable view of mHealth, with a majority stating they either intend to continue using mHealth following the COVID-19 pandemic or would look further into mHealth technologies.

### Provider Demographics

We had a broad representation of specialties and experience levels in our responses. The most common specialties were family medicine, surgery, pediatrics, and physical and occupational therapy. The survey respondents averaged 19 years of experience practicing medicine.

### Clinician Adoption

Clinician adoption of mHealth varied by role and specialty. Doctoral-level providers reported higher mHealth use on their own devices compared with other providers, with 65% (n/N=154/237) and 46% (n/N=138/300) adoption (*P*<.001), respectively. Among mHealth users, doctoral-level providers used these apps as an informational resource at a higher rate (131/154, 85.1% vs 98/138, 71%; *P*=.005). Compared with midtenure providers (16-20 years of experience, 51/81, 63% mHealth adoption), mHealth adoption levels were reported to be lower among more experienced providers (34/72, 47.2% for 21-25 years of experience; 29/64, 45.3% for 26-30 years; and 43/84, 51.2% for >30 years), and similar among less experienced providers (52/95, 54.7% for 0-5 years of experience; 43/73, 58.9% for 6-10 years; and 39/64, 60.9% for 11-15 years). mHealth use with patients was similar between doctoral-level and other providers (84/237, 35.4% vs 110/300, 36.7%; *P*=.77) and across the range of years of experience (33/95, 34.7% for 0-5 years of experience; 27/73, 37% for 6-10 years; 22/64, 34.4% for 11-15 years; 29/81, 35.8% for 16-20 years; 34/72, 47.2% for 21-25 years; 24/64, 37.5% for 26-30 years; and 29/84, 34.5% for >30 years; *P*=.63). Notably, compared with family medicine with 48.4% (n/N=31/64) mHealth use with patients, 3 specialties reported use of ≤30% (Cancer Care and Research, 6/27, 22.2%, *P*=.04 vs family medicine; Cardiology, 7/29, 24.1%, *P*=.05; Surgery, 14/60, 23.3%, *P*=.007), while psychiatry and psychology reported 78.3% (n/N=18/23) adoption, significantly higher than family medicine (*P*=.005). No important differences in reported mHealth use with patients were observed regarding diet and nutrition tracking, weight management, dental reminders, direct communication with the patient’s care team, and medication reminders. Not surprisingly, psychiatry and psychology reported use of mHealth more frequently for mood and depressive symptom monitoring (12/18, 66.7% vs ≤7% for all other specialties that responded to this question [no responses in Cancer Care and Research, Cardiology, OB/GYN, Physical and Occupational Therapy, and Surgery; 1/24, 4.2% in Pediatrics; and 4/65, 6.2% in other specialties], *P*=.001 vs family medicine, 2/31, 6.4%) and sleep tracking (7/18, 38.9% vs ≤16.7% for all other specialties that responded to this question [no responses in Cardiology, OB/GYN and Physical and Occupational Therapy; 1/6, 16.7% in Cancer Care and Research; 2/24, 8.3% in Pediatrics; 1/14, 7.1% in Surgery; and 4/65, 6.2% in other specialties], *P*=.02 vs family medicine, 2/31, 6.4%). Physical and occupational therapy, cardiology, and psychiatry and psychology reported substantially higher mHealth use with patients for informational and educational purposes (12/17, 70.6%; 4/7, 57.1%; and 10/18, 55.6%, with *P*=.002, .048, and .02 vs family medicine, 7/31, 22.6%).

### Clinicians’ Perceived Barriers Category 1—Personal (Clinician)

Overall, providers reported lack of knowledge about mHealth technologies (293/556, 52.7% for themselves; 319/556, 57.4% in their perceptions regarding other clinicians) and lack of time (201/556, 36.2% and 283/556, 50.9%) as the primary personal barriers. Insufficient levels of patient internet access were also a commonly cited concern (171/556, 30.8% and 163/556, 29.3%). We found relatively few differences between provider roles and specialties regarding personal barriers to mHealth adoption. Doctoral-level providers cited a greater number of financial barriers surrounding a lack of value in mHealth technologies (29/237, 12.2% vs 12/300, 4%, *P*<.001 for themselves; 44/237, 18.6% vs 26/300, 8.7%, *P*=.001 in their perceptions regarding other clinicians), insufficient reimbursement options (27/237, 11.4% vs 14/300, 4.7%; *P*=.005 for themselves), and mHealth technologies not being worth the cost of adoption (22/237, 9.3% vs 7/300, 2.3%; *P*=.001 for themselves). With respect to their perceptions regarding other clinicians, cancer care and research providers reported a lack of communication between providers at a substantially higher rate than all other specialties (12/27, 44.4% vs 11.7%-33.3% [5/29, 17.2% in Cardiology; 9/27, 33.3% in OB/GYN; 9/47, 19.1% in Pediatrics; 6/42, 14.3% in Physical and Occupational Therapy; 5/23, 21.7% in Psychiatry and Psychology; 7/60, 11.7% in Surgery; and 45/214, 21% in other specialties] *P*=.02 vs family medicine [13/64, 20.3%]). Furthermore, regarding their perceptions of other clinicians, OB and GYN and pediatrics providers reported a lack of knowledge about mHealth technologies at rates that exceeded all other specialties (21/27, 77.8% and 37/47, 78.7% vs 45%-71.4% [13/27, 48.1% in Cancer Care and Research; 20/29, 69% in Cardiology; 30/42, 71.4% in Physical and Occupational Therapy; 13/23, 56.5% in Psychiatry and Psychology; 27/60, 45% in Surgery; and 110/214, 51.4% in other specialties], *P*=.03 and .01 vs family medicine [36/64, 56.3%]). Interestingly, the only self-perceived barrier that was modified by years of experience was the lack of reliable internet access (*P*=.02 for the overall effect). With the exception of relatively new providers (0-5 years of experience; 22/95, 23.2% of these providers reported this concern), providers in age groups with ≤20 years of experience (31/73, 42.5% with 6-10 years of experience; 24/64, 37.5% with 11-15 years; and 33/81, 40.7% with 16-20 years) reported higher rates of this concern than those in age groups with >20 years of experience (15/72, 20.8% with 21-25 years of experience; 18/64, 28.1% with 26-30 years; and 23/84, 27.4% with >30 years).

### Clinicians’ Perceived Barriers Category 2—Patient

Survey respondents reported substantial perceived patient concerns relating to mHealth technologies being too complicated (371/556, 66.7%), lack of access to mHealth technologies (327/556, 58.8%), poor delivery mechanisms (eg, cell service or internet coverage, 252/556, 45.3%), and privacy concerns (207/556, 37.2%). These perceptions did not differ meaningfully by provider type, specialty, or years of experience, with the exception that privacy concerns were more prevalent in doctoral-level providers (105/237, 44.3% vs 93/300, 31%; *P*=.002).

### Clinicians’ Perceived Barriers Category 3—Organizational

The most prevalent organizational barriers perceived by providers were concerns related to confidentiality (313/556, 56.3%) and that mHealth technologies were too complicated for patients (288/556, 51.8%). Confidentiality concerns differed meaningfully by provider type (149/237, 62.9% and 154/300, 51.3% for doctoral-level vs other providers, *P*=.007), specialty (*P*<.001 for the overall specialty effect; 37/47, 78.7% vs 36/64, 56.3% for pediatrics vs family medicine, *P*=.01), and years of experience (*P*=.03 for the overall effect; no specific trend across age groups). Privacy concerns (168/556, 30.2% prevalence) varied only by years of experience (*P*=.006 for the overall effect; no specific trend across age groups).

### COVID-19 and Anticipated mHealth Adoption

**Figure 2:**
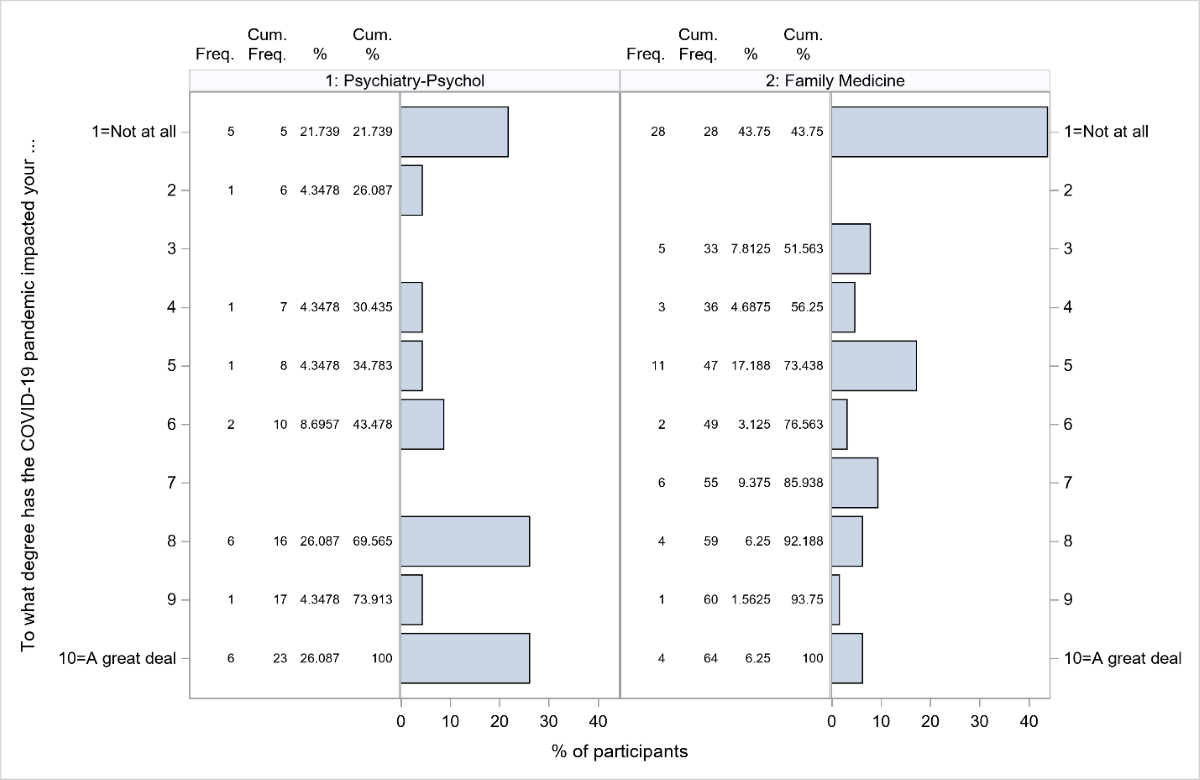
Response distributions for psychiatry and psychology and family medicine for the question “To what degree has the COVID-19 pandemic impacted your mHealth adoption?”. mHealth: mobile health.

**Figure 3:**
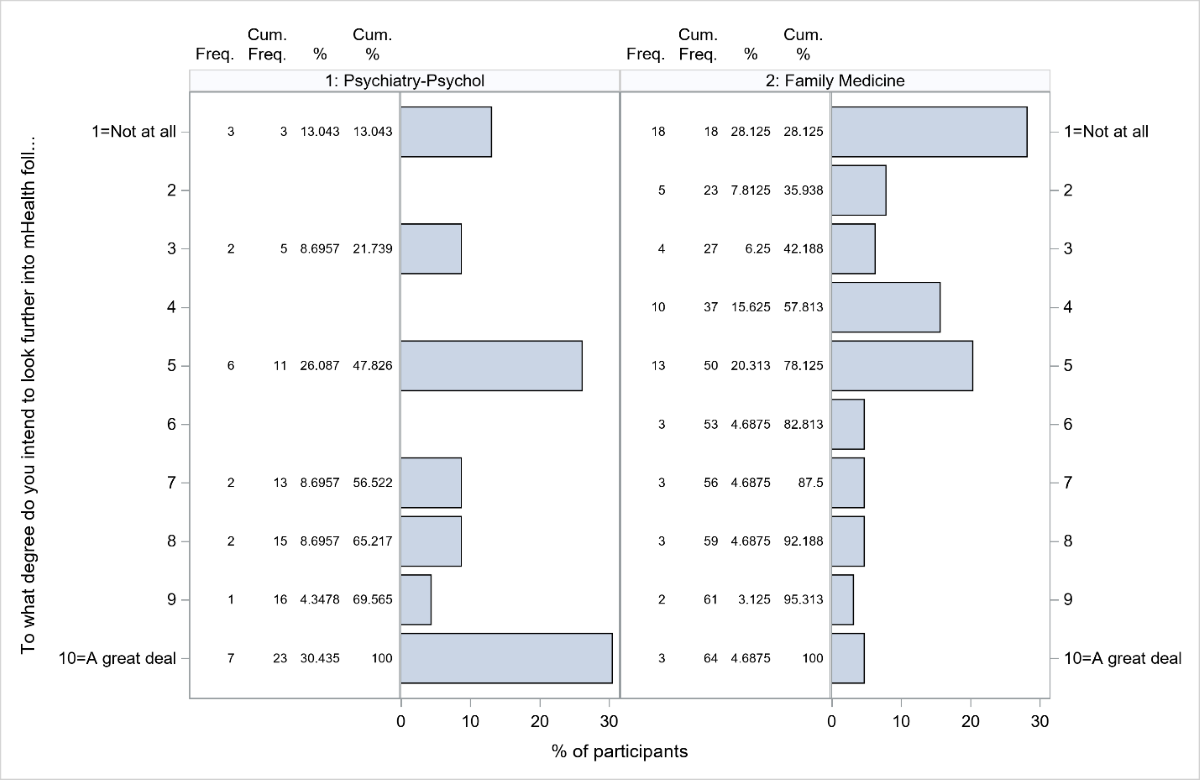
Response distributions for psychiatry and psychology and family medicine for the question “To what degree do you intend to look further into mHealth following the resumption of normal MCHS activities?” MCHS: Marshfield Clinic Health System; mHealth: mobile health.

When providers were asked to what degree (1) the COVID-19 pandemic impacted their mHealth adoption and (2) they intend to look further into mHealth following the resumption of normal MCHS activities, meaningful differences were detected only between provider specialties (*P*=.02 and .001 for the overall specialty effects, respectively). These differences were driven by psychiatry and psychology providers, who reported higher scores (10-point Likert scale, where 1=not at all and 10=a great deal) on both survey questions (*P*=.002 and .002 vs family medicine; Figures 2 and 3).

## Discussion

### Principal Findings

The varied responses and rate of mHealth use across provider roles and specialties emphasize the variety and task-specific role mHealth can have in a health system. Some specialties, such as psychology and psychiatry, showed high rates of adoption for specific tasks such as mood and sleep tracking; however, no other specialties reported substantially greater mHealth adoption compared with the reference to family medicine. If looking to increase mHealth use across a health system, leadership should consider identifying specific tasks or poorly performing metrics that mHealth could potentially improve upon.

Our survey grouped potential barriers into 3 levels (provider, patient, and organizational) in line with past qualitative findings [13]. Providers’ self-barriers encompassed themes of lack of knowledge about mHealth technologies, lack of time, and lack of patients’ access to the internet. Commonly reported barriers relating to both patients and the organization were mHealth technologies being too complicated and concerns related to privacy. The predominant organizational barrier was confidentiality concerns, whereas lack of access (cell phone coverage, internet, and mHealth technology in general) was a frequently perceived barrier for patients.

It is understandable that health care providers feel overwhelmed, with the top barrier to mHealth use being a lack of time and information. These technologies evolve at incredible speeds. How might one stay abreast of the scientific and technological advances of mHealth technologies? Even in the peer-reviewed literature, which can take months or years to publish, we witness an overwhelming ocean of information. At the time of this writing, a Google Scholar search of “mHealth” papers since 2020 (January 1, 2020, to April 5, 2024) yielded nearly 25,000 results and nearly 8000 results since January 1, 2024.

The pace of emerging and simultaneously retiring technologies remains a substantial barrier across many mHealth studies. A typical full-scale trial to evaluate a mHealth initiative lasts more than 5 years from recruitment, during which time many changes within the pertinent technologies will occur or be superseded entirely [20]. Consequentially, many trials are reduced in scope, hindering true evaluation and understanding of the prospect’s long-term value.

Years of technological ambition surrounding deep machine learning and voluminous data sets reached fruition in the 2020s with the advent of widely accessible artificial intelligence (AI) apps. These AI-powered breakthroughs have impacted nearly every industry, including medicine, in ways that are still in the infancy of exploration. By digitally processing millions of training samples, including imagery, transcripts, audio recordings, and academic papers, sophisticated computer algorithms have reached new potential in data analysis and user reactivity [21]. What once required thousands of hours and access to prohibitively expensive data centers to compute is now within a finger’s reach from any consumer phone or computer.

Leveraging computer-assisted workflows to automate tasks is not a new concept in the medical world. Health care organizations have spent decades exploring increasingly advanced forms of speech recognition software to facilitate medical transcriptions, among other areas of automation [22]. The latest groundbreaking strides in these efforts come in the form of OpenAI’s ChatGPT and associated tool sets [23]. A recent study hypothesized more than 130 different ways ChatGPT could positively benefit both patients and doctors in the foreseeable future, including education, prediction support, prevention of medical errors, record-keeping, and continual clinical assistance [24].

However, many analysts warn that such tool sets—when used in isolation without a human consultant—can yield bad data or other repercussions not yet realized. The most prevalent example of these dangers is how AI modeling is prone to hallucinations, in which the chatbot may return seemingly factual and confident responses but uses nonexistent citations or made-up passages due to anomalies in its training data and other limitations [25,26].

### Limitations

The response rate to our mHealth survey was 38% (n/N=916/2410), with a further completion rate of 60.7% (n/N=556/916). It is possible that response bias was present, potentially skewing toward clinicians who have an interest in mHealth technologies. While this response rate is moderately high compared with other surveys of providers, the topic of mHealth being mentioned foremost in the survey invitation may have resulted in an overestimation of mHealth use and intentions.

Notably, MCHS, along with many other health care organizations at the time, was struggling due to the COVID-19 pandemic during our survey timeframe, with rolling temporary furloughs throughout the health system. This limited our possible response rate and created uncertainty in the accuracy and complete capture of our sample. However, our survey was open for 6 weeks with multiple reminder emails sent out, theoretically limiting this effect. Nevertheless, biases in responses may remain due to the work environment and shifting priorities surrounding the COVID-19 pandemic. A future resurveying of providers would help characterize these possible impacts.

### Conclusions

Health systems should continue to evaluate mHealth adoption, and more formally and proactively investigate innovative solutions. Consulting with patient safety and legal departments regarding the use of mHealth apps is crucial, as quality clinical outcomes are not often in correlation with popularity ratings on app stores [27]. If a mHealth tool is deemed to be a valuable tool for a hospital or health system, leadership should work toward identifying specific options and methods to address health outcomes and work toward simple and concise implementations to improve adoption and patient outcomes. The American Medical Association provides occasional reports and guidelines surrounding mHealth best practices, but does not have an official lobbying body, with more focus on telehealth [28-30]. The US Department of Health and Human Services provides resources for mHealth developers; however, these are primarily focused on privacy and confidentiality and are of little relevance to providers [31].

This study is arguably a foundational and necessary step in assessing a health system’s status and potential for mHealth adoption. Further research and continued partnership with advisors and stakeholders will be needed if the health system hopes to more formally integrate mHealth technologies into rural health care.

## Abbreviations

AI: artificial intelligence
MCHS: Marshfield Clinic Health System
mHealth: mobile health

## References

[1] Park YT (2016). Emerging new era of mobile health technologies. Healthc Inform Res.

[2] Alam MZ, Proteek SM, Hoque I (2023). A systematic literature review on mHealth related research during the COVID-19 outbreak. Health Educ.

[3] Alam MMD, Alam MZ, Rahman SA, Taghizadeh SK (2021). Factors influencing mHealth adoption and its impact on mental well-being during COVID-19 pandemic: a SEM-ANN approach. J Biomed Inform.

[4] Gagnon MP, Ngangue P, Payne-Gagnon J, Desmartis M (2016). m-Health adoption by healthcare professionals: a systematic review. J Am Med Inform Assoc.

[5] (2023). Market share of mobile operating systems worldwide from 2009 to 2023, by quarter. Statista.

[6] (2023). Network coverage outlook and mobility report. Ericsson.

[7] Govoruhina A, Nikiforova A (2022). Digital health shopping assistant with react native: a simple technological solution to a complex health problem.

[8] Fentaw AE (2020). Cross platform mobile application development: a comparison study of react native vs flutter. JYX Digital Repository.

[9] (2023). Number of connected wearable devices worldwide from 2016 to 2022. Statista.

[10] Niwa M, Kodama K, Sengoku S (2022). mHealth as a component of next-generation health care. Mobile Health (mHealth): Rethinking Innovation Management to Harmonize AI and Social Design.

[11] (2023). Non-invasive blood glucose monitoring closer as Apple hits major milestone. Diabaetes.co.uk.

[12] Sheng A, Lin L, Zhu J, Zhuang J, Li J, Chang L, Cheng H (2021). Micro/nanodevices for assessment and treatment in stomatology and ophthalmology. Microsyst Nanoeng.

[13] Khatun F, Heywood AE, Ray PK, Hanifi SM, Bhuiya A, Liaw S (2015). Determinants of readiness to adopt mHealth in a rural community of Bangladesh. Int J Med Inform.

[14] Weichelt B, Bendixsen C, Patrick T (2019). A model for assessing necessary conditions for rural health care's mobile health readiness: qualitative assessment of clinician-perceived barriers. JMIR Mhealth Uhealth.

[15] Shimpi N, Glurich I, Maybury C, Wang MQ, Hashimoto K, Acharya A, Horowitz AM (2021). Knowledge, attitudes, behaviors of women related to pregnancy, and early childhood caries prevention: a cross-sectional pilot study. J Prim Care Community Health.

[16] (2023). About marshfield clinic health system. Marshfield Clinic Health System.

[17] McNutt LA, Wu C, Xue X, Hafner JP (2003). Estimating the relative risk in cohort studies and clinical trials of common outcomes. Am J Epidemiol.

[18] Kruskal WH, Wallis WA (1952). Use of ranks in one-criterion variance analysis. J Am Stat Assoc.

[19] Hollander M, Wolfe DA (1999). Nonparametric Statistical Methods.

[20] Kumar S, Nilsen WJ, Abernethy A, Atienza A, Patrick K, Pavel M, Riley WT, Shar A, Spring B, Spruijt-Metz D, Hedeker D, Honavar V, Kravitz R, Lefebvre RC, Mohr DC, Murphy SA, Quinn C, Shusterman V, Swendeman D (2013). Mobile health technology evaluation: the mHealth evidence workshop. Am J Prev Med.

[21] Glock P, von Alemann S, Waltl B, Schindler D, Jacob K, Strathausen R (2022). The paradigm shift in AI: from human labor to humane creativity. Liquid Legal – Humanization and the Law.

[22] Borowitz SM (1999). Computer-based speech recognition as a replacement for medical transcription. Pediatr Res.

[23] Hughes A (2023). ChatGPT: everything you need to know about OpenAI's GPT-4 tool. BBC Science Focus Magazine.

[24] Javaid M, Haleem A, Singh RP (2023). ChatGPT for healthcare services: an emerging stage for an innovative perspective. BenchCouncil Trans Benchmarks Stand Eval.

[25] Athaluri SA, Manthena SV, Kesapragada VSRKM, Yarlagadda V, Dave T, Duddumpudi RTS (2023). Exploring the boundaries of reality: investigating the phenomenon of artificial intelligence hallucination in scientific writing through ChatGPT references. Cureus.

[26] Brereton D (2023). Bing AI can't be trusted. DKB Blog.

[27] Wyatt JC (2018). How can clinicians, specialty societies and others evaluate and improve the quality of apps for patient use?. BMC Med.

[28] (2016). AMA adopts principles to promote safe, effective mHealth applications. American Medical Association.

[29] (2022). AMA's advocacy efforts push for permanent telehealth advancements. American Medical Association.

[30] (2023). Comprehensive telehealth reform is critical to the future of health care. American Medical Association.

[31] (2022). Resources for mobile health apps developers. US Department of Health and Human Services.

